# Supplementary material for: A rich conformational palette underlies human CaV2.1-channel availability
Source: Nat Commun. 2025 Apr 23;16:3815. doi: 10.1038/s41467-025-58884-2 (PMC12019389; doi:10.1038/s41467-025-58884-2)
Supplement: Supplementary file 1 — Supplementary Information [file 41467_2025_58884_MOESM1_ESM.pdf]

Supplementary Materials for:

A Rich Conformational Palette Underlies Human $Ca_V2.1$-Channel Availability

2025, *Nature Communications*

Author list

Kaiqian Wang[1], Michelle Nilsson[1], Marina Angelini[2], Riccardo Olcese[2,3], Fredrik Elinder[1,4], Antonios Pantazis[1,5]*

Affiliations

[1]Division of Cell and Neurobiology, Department of Biomedical and Clinical Sciences, Linköping University; SE-581 85 Linköping, Sweden

[2]Department of Anesthesiology and Perioperative Medicine, David Geffen School of Medicine, University of California, Los Angeles; Los Angeles, CA 90095, USA

[3]Department of Physiology, David Geffen School of Medicine, University of California, Los Angeles; Los Angeles, CA 90095, USA

[4]Science for Life Laboratory, Linköping University; SE-581 85 Linköping, Sweden

[5]Wallenberg Center for Molecular Medicine, Linköping University; SE-581 85 Linköping, Sweden

*Corresponding author. Email: antonios.pantazis@liu.se

This PDF file includes:

Figs. S1, S2, S3
Tables S1, S2, S3

Other Supplementary Materials for this manuscript include the following:

Supplementary Movie 1

FIGURE S1

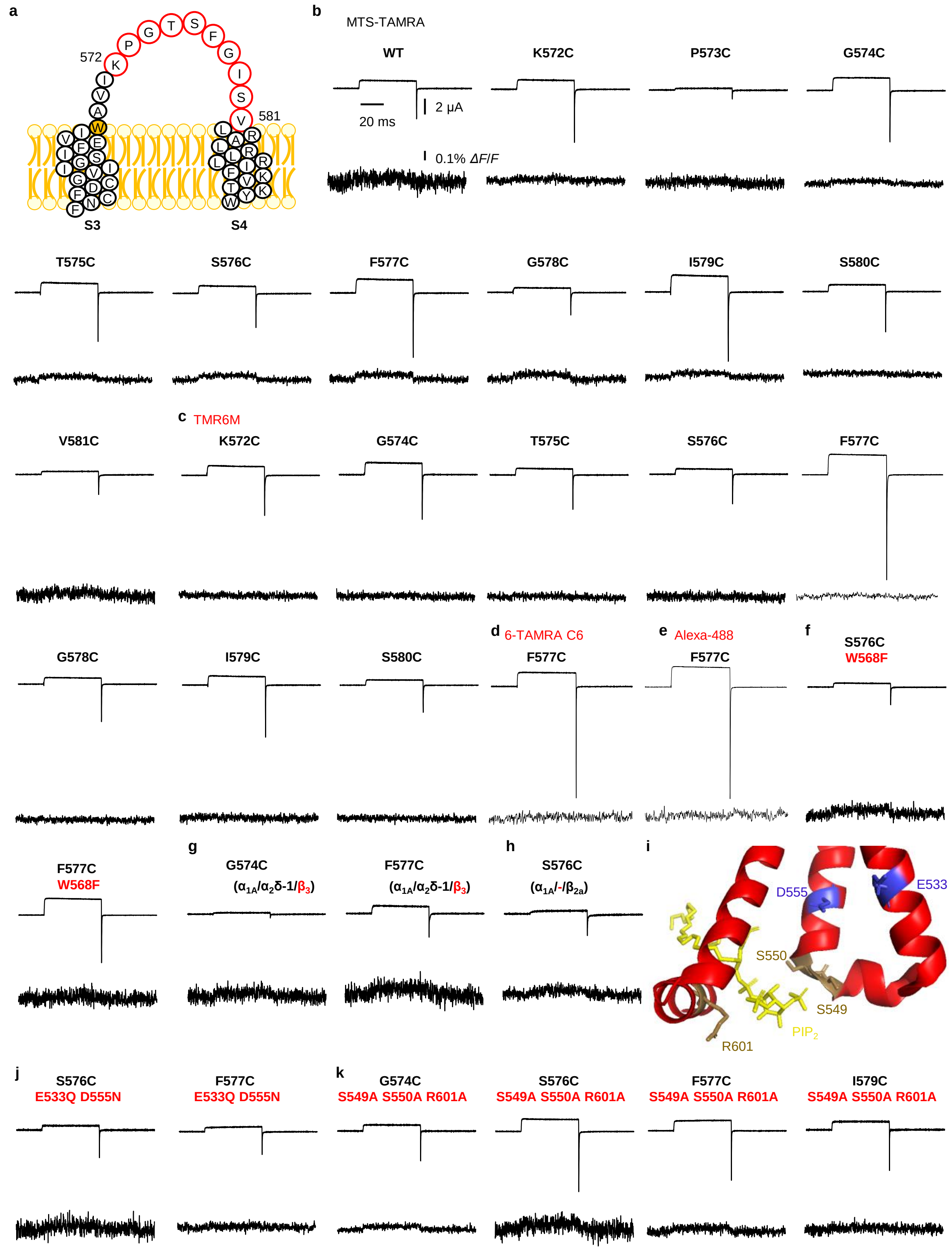

a
572
K P G T S F G I S V
581
S3
S4
b
MTS-TAMRA
WT
K572C
P573C
G574C
20 ms
2 μA
0.1% ΔF/F
T575C
S576C
F577C
G578C
I579C
S580C
V581C
c
TMR6M
K572C
G574C
T575C
S576C
F577C
G578C
I579C
S580C
d
6-TAMRA C6
F577C
e
Alexa-488
F577C
f
S576C
W568F
F577C
W568F
g
G574C
(α1A/α2δ-1/β3)
F577C
(α1A/α2δ-1/β3)
h
S576C
(α1A/-/β2a)
i
D555
E533
S550
S549
PIP2
R601
j
S576C
E533Q D555N
F577C
E533Q D555N
k
G574C
S549A S550A R601A
S576C
S549A S550A R601A
F577C
S549A S550A R601A
I579C
S549A S550A R601A

Fig. S1. No voltage-dependent activation of $Ca_V2.1$ VSD-II is detected. (a) Snake plot of the S3-S4 segment, and extracellular linker, of $Ca_V2.1$ VSD-II. Positions tested by VCF are indicated by red circles. **(b)** Current and fluorescence traces from MTS-TAMRA-labelled $Ca_V2.1$ channel complexes (α_{1A} construct indicated + $\alpha_2\delta$-1 + β_{2a}) in response to a voltage step from −80 mV to 80 mV. **(c-e)** As in (b) for channels labeled with fluorophores TMR6M, 6-TAMRA C6 maleimide or Alexa-488 maleimide, respectively. **(f-h)** As in (b), using channels with a substituted Trp in S3, co-expressed with the β_3 subunit, or lacking the $\alpha_2\delta$-1 subunit, respectively. **(i)** Magnified view of the PIP_2-binding site in VSD-II (PDB: 8X90). PIP_2-binding residues (sand-coloured) and counter-charge residues (blue) are indicated. **(j,k)** As in (b), using channels lacking two counter-charge residues in S2-S3, or the PIP_2 binding residues, respectively. Despite extensive efforts, no fluorescence deflections are observed from $Ca_V2.1$ channels fluorescently labelled in VSD-II in response to membrane depolarization.

FIGURE S2

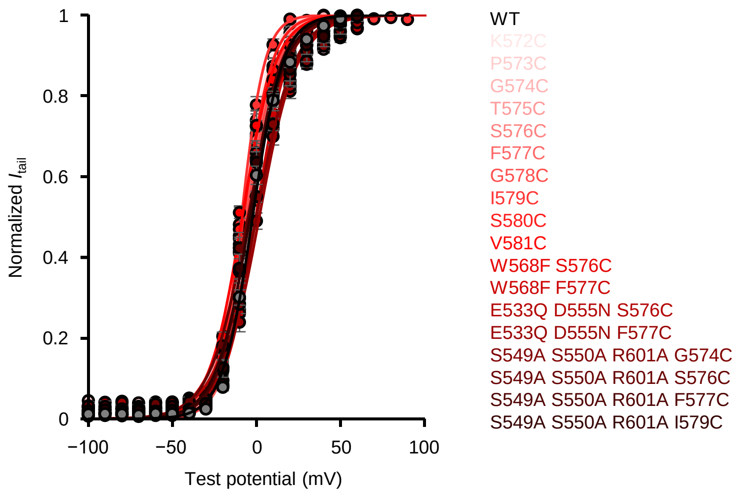

**α_{1A} construct**		$V_{0.5}$ (mV)	z (e_0)	n (cells)
Wild-type		−2.2±0.9	2.9±0.1	12
Cys substitutions for VCF	K572C	−7.9±1.0	3.3±0.1	7
	P573C	0.6±0.5	2.1±0.1	20
	G574C	−6.4±1.1	3.3±0.2	9
	T575C	−6.5±0.9	2.9±0.1	10
	S576C	−6.6±0.8	2.7±0.1	10
	F577C	−2.3±0.6	3.1±0.1	26
	G578C	−6.1±0.6	2.7±0.1	8
	I579C	−8.9±0.9	3.8±0.1	8
	S580C	−7.4±1.9	3.2±0.3	6
	V581C	−8.4±0.5	2.6±0.1	11
Removal of Trp at the top of S3 (and Cys substitutions)	W568F S576C	−4.9±1.4	2.3±0.1	8
	W568F F577C	−0.4±0.3	2.7±0.1	11
Removal of counter-charges (and Cys substitutions)	E533Q D555N S576C	−0.1±0.6	2.1±0.1	10
	E533Q D555N F577C	1.7±1.0	2.3±0.1	10
Removal of PIP_2 binding site (and Cys substitutions)	S549A S550A R601A G574C	0.1±0.7	2.3±0.1	11
	S549A S550A R601A S576C	−4.9±1.7	2.2±0.1	4
	S549A S550A R601A F577C	−4.0±0.8	2.8±0.1	20
	S549A S550A R601A I579C	−3.5±1.6	2.5±0.1	6

Fig. S2. Mutations in VSD-II have minimal effects on CaV2.1 voltage-dependent opening. Normalized tail current-voltage relationships were fit to a Boltzmann distribution (eq.1). Errors are S.E.M.

FIGURE S3

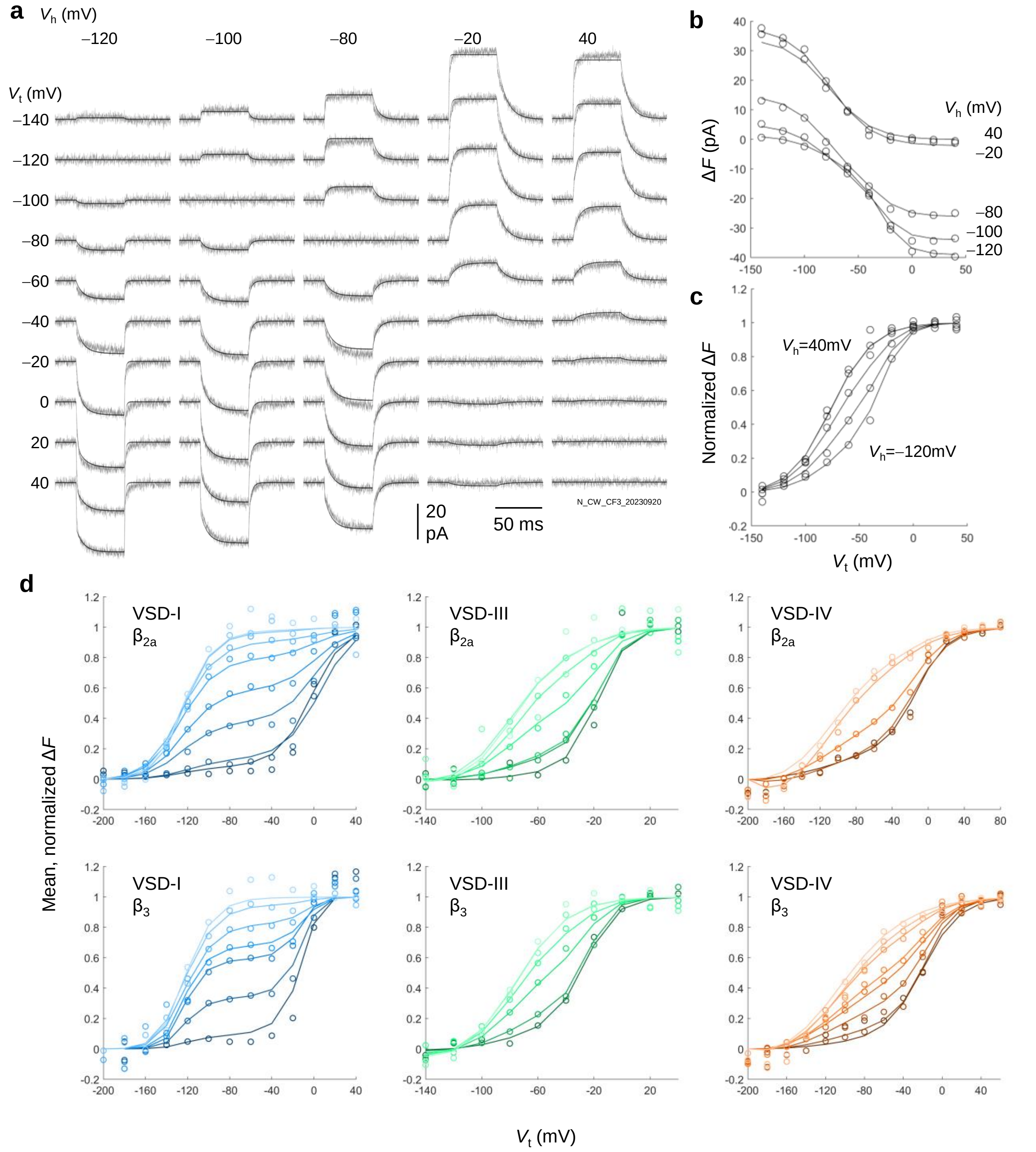

Fig. S3. 4-state model fitting. (a) Fitting of Δ*F* traces in a representative cell expressing VSD-III-labeled $Ca_V2.1$-channels with β_3. VSD-III activation was probed with test potentials (V_t) from –140 to 40 mV over the holding potentials (V_h) indicated, from –120 to 40 mV. Grey traces are the Δ*F* data; black lines are the model output (eq.14). A total of 14 cells from this condition (VSD-III, β_3) were fit thus, and 81 cells across all conditions. **(b,c)** Model fits (black curves) over the raw (b) and normalized (c) Δ*F* (open circles) from the cell in (a). **(d)** Mean model fits (curves) and mean, normalized Δ*F* (open circles). Parameters are in table S2. V_h, from darker to

lighter shade (in mV): VSD-I, β_{2a}: −120, −100, −80, −60, −40, −20, 0, 40; VSD-I, β_3: −120, −100, −90, −80, −60, −40, 40; VSD-III, β_{2a}: −160, −120, −100, −80, −60, −40, 40; VSD-III, β_3: −140, −120, −100, −80, −20, 40; VSD-IV, β_{2a}: −120, −100, −80, −20, 40; VSD-IV, β_3: −160, −120, −100, −90, −80, −60, −40, 40.

TABLE S1

VSD-I β2a (Fig.3f)								
V_h (mV)	−120	−100	−80	−60	−40	−20	0	40
F_1 (%)	98.5 ± 1.04	92.3 ± 1.23	69.2 ± 2.37	60.0 ± 2.21	38.3 ± 3.45	9.66 ± 1.75	0.0175 ± 0.0175	0.489 ± 0.343
$V_{0.5\_1}$ (mV)	−0.996 ± 1.09	2.62 ± 1.56	4.31 ± 0.966	7.02 ± 1.54	7.39 ± 0.951	8.00 ± 0.899	5.18 ± 1.96	6.85 ± 1.24
z_1 (e_0)	2.40 ± 0.157	2.53 ± 0.138	2.36 ± 0.103	2.11 ± 0.0987	2.12 ± 0.124	2.10 ± 0.124	1.87 ± 0.185	1.82 ± 0.0847
$V_{0.5\_2}$ (mV)	−123 ± 3.10	−120 ± 3.09	−123 ± 1.76	−123 ± 2.74	−121 ± 1.80	−122 ± 1.19	−123 ± 0.923	−125 ± 1.65
z_2 (e_0)	2.37 ± 0.177	2.53 ± 0.138	2.36 ± 0.103	2.11 ± 0.0987	2.12 ± 0.124	2.10 ± 0.124	1.90 ± 0.0979	1.81 ± 0.0386
n (cells)	8	10	24	12	11	11	9	17

VSD-I β3 (Fig.3i)							
V_h (mV)	−120	−100	−90	−80	−60	−40	40
F_1 (%)	97.0 ± 1.70	73.4 ± 4.61	46.8 ± 6.76	33.1 ± 3.33	17.6 ± 3.41	1.06 ± 1.06	0.00 ± 0.00
$V_{0.5\_1}$ (mV)	−5.47 ± 0.920	−5.72 ± 0.884	−5.25 ± 1.44	−5.27 ± 0.629	−5.08 ± 0.865	−5.00 ± 1.04	-
z_1 (e_0)	3.39 ± 0.147	3.44 ± 0.145	3.25 ± 0.173	4.08 ± 0.228	4.67 ± 0.289	5.04 ± 0.258	-
$V_{0.5\_2}$ (mV)	−126 ± 2.00	−126 ± 1.84	−125 ± 2.17	−124 ± 1.35	−124 ± 2.32	−121 ± 0.783	−121 ± 1.28
z_2 (e_0)	1.70 ± 0.110	1.70 ± 0.102	1.73 ± 0.110	1.75 ± 0.0760	1.57 ± 0.0881	1.60 ± 0.0873	1.76 ± 0.0675
n (cells)	12	13	6	20	11	7	9

VSD-III β2a (Fig.4a)							
V_h (mV)	−160	−120	−100	−80	−60	−40	40
F_1 (%)	93.0	87.7 ± 2.14	77.7 ± 4.77	38.1 ± 3.08	25.9 ± 4.37	26.0 ± 4.05	0.233 ± 0.233
$V_{0.5\_1}$ (mV)	−13.1	−16.2 ± 0.980	−15.7 ± 1.33	−15.9 ± 0.941	−18.3 ± 1.15	−12.5 ± 1.75	−15.9 ± 0.941
z_1 (e_0)	2.87	3.33 ± 0.184	3.59 ± 0.236	3.28 ± 0.168	3.30 ± 0.26	3.95 ± 0.377	3.28 ± 0.168
$V_{0.5\_2}$ (mV)	−76.4	−72.3 ± 1.14	−71.5 ± 1.50	−72.7 ± 1.14	−73.1 ± 2.43	−69.5 ± 1.34	−72.7 ± 1.14
z_2 (e_0)	1.82	1.84 ± 0.131	2.09 ± 0.158	1.84 ± 0.120	± 1.95 0.110	2.25 ± 0.328	1.84 ± 0.120
n (cells)	2	17	11	19	6	5	19

VSD-III β3 (Fig.4d)					
V_h (mV)	−120	−100	−80	−20	40
F_1 (%)	70.1 ± 2.62	33.4 ± 1.77	10.9 ± 1.90	0.00 ± 0.00	0.00 ± 0.00
$V_{0.5\_1}$ (mV)	−25.4 ± 1.56	−26.0 ± 1.60	−26.2 ± 1.64	-	-
z_1 (e_0)	2.77 ± 0.175	2.69 ± 0.167	2.70 ± 0.178	-	-
$V_{0.5\_2}$ (mV)	−77.7 ± 1.91	−78.2 ± 2.03	−78.9 ± 2.16	−76.7 ± 1.72	−78.3 ± 2.00
z_2 (e_0)	1.45 ± 0.0427	1.43 ± 0.0441	1.45 ± 0.0394	1.46 ± 0.0844	1.45 ± 0.0469
n (cells)	12	11	13	6	11

VSD-IV β2a (Fig.4g)						
V_h (mV)	−120	−100	−80	−60	−20	40
$F1$ (%)	55.4 ± 2.94	52.1 ± 5.39	37.6 ± 2.26	32.2 ± 1.59	2.80 ± 1.43	0.00 ± 0.00
$V_{0.5\_1}$ (mV)	−9.58 ± 0.922	−7.73 ± 0.917	−8.59 ± 0.737	−9.63 ± 1.22	−7.96 ± 1.10	-
z_1 (e_0)	2.97 ± 0.183	3.57 ± 0.324	3.17 ± 0.189	2.48 ± 0.0905	3.68 ± 0.223	-
$V_{0.5\_2}$ (mV)	−99.0 ± 2.65	−93.0 ± 2.65	−97.2 ± 1.95	−102 ± 2.36	−93.9 ± 1.63	−99.2 ± 2.38
z_2 (e_0)	0.656 ± 0.0318	0.617 ± 0.0783	0.665 ± 0.0267	0.641 ± 0.0414	0.679 ± 0.0597	0.695 ± 0.0292
n (cells)	18	4	26	4	7	19

VSD-IV β3 (Fig.4j)								
V_h (mV)	−160	−120	−100	−90	−80	−60	−40	40
$F1$ (%)	69.2 ± 1.52	62.4 ± 2.48	50.6 ± 4.07	39.1 ± 3.36	27.6 ± 2.04	12.8 ± 3.27	4.02 ± 2.28	0.00 ± 0.00
$V_{0.5\_1}$ (mV)	−14.1 ± 0.369	−15.3 ± 0.701	−16.1 ± 0.822	−14.7 ± 0.682	−15.0 ± 0.533	−16.1 ± 0.822	−14.4 ± 1.14	-
z_1 (e_0)	2.91 ± 0.275	2.98 ± 0.191	3.24 ± 0.251	2.64 ± 0.191	2.97 ± 0.153	3.24 ± 0.251	2.68 ± 0.253	-
$V_{0.5\_2}$ (mV)	−102 ± 2.18	−100 ± 0.772	−101 ± 1.23	−101 ± 1.20	−101 ± 0.785	−101 ± 1.23	−99.5 ± 0.714	−101 ± 0.785
z_2 (e_0)	0.787 ± 0.0193	0.773 ± 0.0181	0.763 ± 0.0261	0.780 ± 0.0204	0.776 ± 0.0140	0.763 ± 0.0261	0.784 ± 0.0271	0.776 ± 0.0140
n (cells)	4	11	6	7	15	6	5	15

Table. S1. Conversion Boltzmann parameters. Equation 3 was used. Errors are S.E.M.

TABLE S2

β_{2a} — **VSD-I** (*n*=13 cells) / **VSD-III** (*n*=16) / **VSD-IV** (*n*=14)

R1↔A1 (mode 1 activation / deactivation)

parameter	notes	VSD-I mean	VSD-I low CI 95%	VSD-I high CI 95%	VSD-III mean	VSD-III low CI 95%	VSD-III high CI 95%	VSD-IV mean	VSD-IV low CI 95%	VSD-IV high CI 95%
k_{eq} (s^{-1})	1	39.1	16.6	66.4	159	137	179	195	175	215
V_{eq} (mV)		4.42	–4.73	11.3	–18.3	–19.6	–17.3	–13.5	–18.7	–11.6
z (e_0)		1.52	1.41	1.64	2.44	2.30	2.60	1.39	1.31	1.49
β		0.769	0.693	0.863	0.721	0.695	0.744	0.626	0.582	0.649

R2↔A2 (mode 2 activation / deactivation)

parameter	notes	VSD-I mean	VSD-I low CI 95%	VSD-I high CI 95%	VSD-III mean	VSD-III low CI 95%	VSD-III high CI 95%	VSD-IV mean	VSD-IV low CI 95%	VSD-IV high CI 95%
k_{eq} (s^{-1})	1	14.8	12.6	16.9	87.4	72.0	96.2	138	116	163
V_{eq} (mV)		–123	–126	–121	–78.7	–81.7	–76.6	–119	–131	–111
z (e_0)	2	1.52	1.41	1.64	1.41	1.30	1.54	0.741	0.661	0.813
β		0.309	0.294	0.320	0.0374	0.0209	0.0624	0.655	0.585	0.762

R1↔R2 (resting-state conversion / recovery)

parameter	notes	VSD-I mean	VSD-I low CI 95%	VSD-I high CI 95%	VSD-III mean	VSD-III low CI 95%	VSD-III high CI 95%	VSD-IV mean	VSD-IV low CI 95%	VSD-IV high CI 95%
k_{con} (s^{-1})	1	0.00425	0.000995	0.0170	1.12	0.594	2.16	0.167	0.0643	0.428
k_{rec} (s^{-1})	1	0.157	0.0376	0.548	0.0688	0.0394	0.130	0.0835	0.0266	0.207
V_{eq} (mV)	3	-	-	-	–65.3	–72.3	–52.8	–16.8	–33.0	1.46
z (e_0)	4	0	0	0	1.03	0.893	1.17	0.652	0.532	0.753
β		-	-	-	0.512	0.369	0.637	0.498	0.310	0.696

A1↔A2 (active-state conversion / recovery)

parameter	notes	VSD-I mean	VSD-I low CI 95%	VSD-I high CI 95%	VSD-III mean	VSD-III low CI 95%	VSD-III high CI 95%	VSD-IV mean	VSD-IV low CI 95%	VSD-IV high CI 95%
k_{con} (s^{-1})	1,5	0.773	0.0880	3.73	0.115	0.0553	0.237	0.0176	0.00551	0.128
k_{rec} (s^{-1})	1	0.0148	0.00202	0.0757	0.000511	0.000258	0.00110	0.000620	0.000198	0.00508
z (e_0)	6	0	0	0	0	0	0	0	0	0

Mode 1↔2 interconversion

parameter	notes	VSD-I mean	VSD-I low CI 95%	VSD-I high CI 95%	VSD-III mean	VSD-III low CI 95%	VSD-III high CI 95%	VSD-IV mean	VSD-IV low CI 95%	VSD-IV high CI 95%
$V_{0.5}$ (mV)	3	–62.2	–67.8	–58.3	–83.1	–86.0	–79.5	–79.2	–83.3	–75.2

Pore opening

parameter	notes	VSD-I mean	VSD-I low CI 95%	VSD-I high CI 95%	VSD-III mean	VSD-III low CI 95%	VSD-III high CI 95%	VSD-IV mean	VSD-IV low CI 95%	VSD-IV high CI 95%
$V_{0.5}$ (mV)	7	8.03	5.97	11.4	–0.0189	–1.52	1.32	–0.674	–1.59	0.481

β_3 — **VSD-I** (*n*=10) / **VSD-III** (*n*=14) / **VSD-IV** (*n*=14)

R1↔A1 (mode 1 activation / deactivation)

parameter	notes	VSD-I mean	VSD-I low CI 95%	VSD-I high CI 95%	VSD-III mean	VSD-III low CI 95%	VSD-III high CI 95%	VSD-IV mean	VSD-IV low CI 95%	VSD-IV high CI 95%
k_{eq} (s^{-1})	1	4.13	2.21	9.06	167	133	205	146	128	197
V_{eq} (mV)		–22.4	–29.9	–14.0	–25.0	–26.4	–23.5	–17.5	–18.7	–16.3
z (e_0)		2.37	2.10	2.62	2.74	2.53	2.91	1.57	1.46	1.68
β		0.790	0.725	0.873	0.806	0.775	0.843	0.708	0.696	0.722

R2↔A2 (mode 2 activation / deactivation)

parameter	notes	VSD-I mean	VSD-I low CI 95%	VSD-I high CI 95%	VSD-III mean	VSD-III low CI 95%	VSD-III high CI 95%	VSD-IV mean	VSD-IV low CI 95%	VSD-IV high CI 95%
k_{eq} (s^{-1})	1	9.33	7.68	10.7	91.1	85.2	99.4	182	168	196
V_{eq} (mV)		–118	–121	–112	–76.8	–79.7	–74.7	–108	–112	–105
z (e_0)	2	2.37	2.11	2.62	1.32	1.24	1.39	0.743	0.705	0.783
β		0.335	0.277	0.417	0.0419	0.0246	0.0594	0.593	0.561	0.630

R1↔R2 (resting-state conversion / recovery)

parameter	notes	VSD-I mean	VSD-I low CI 95%	VSD-I high CI 95%	VSD-III mean	VSD-III low CI 95%	VSD-III high CI 95%	VSD-IV mean	VSD-IV low CI 95%	VSD-IV high CI 95%
k_{con} (s^{-1})	1	0.0121	0.00553	0.0362	0.831	0.461	1.52	0.245	0.160	0.327
k_{rec} (s^{-1})	1	0.217	0.0876	0.550	0.00223	0.00110	0.00407	0.0287	0.0191	0.0395
V_{eq} (mV)	3	-	-	-	–105	–108	–101	–64.3	–71.1	–56.4
z (e_0)	4	0	0	0	1.42	1.24	1.59	0.829	0.746	0.926
β		-	-	-	0.357	0.220	0.540	0.412	0.298	0.584

A1↔A2 (active-state conversion / recovery)

parameter	notes	VSD-I mean	VSD-I low CI 95%	VSD-I high CI 95%	VSD-III mean	VSD-III low CI 95%	VSD-III high CI 95%	VSD-IV mean	VSD-IV low CI 95%	VSD-IV high CI 95%
k_{con} (s^{-1})	1,5	1.39	0.303	3.78	0.232	0.0967	0.463	0.00912	0.00650	0.0133
k_{rec} (s^{-1})	1	0.00344	0.000820	0.0109	0.000169	0.000136	0.000276	0.000133	0.000128	0.000140
z (e_0)	6	0	0	0	0	0	0	0	0	0

Mode 1↔2 interconversion

parameter	notes	VSD-I mean	VSD-I low CI 95%	VSD-I high CI 95%	VSD-III mean	VSD-III low CI 95%	VSD-III high CI 95%	VSD-IV mean	VSD-IV low CI 95%	VSD-IV high CI 95%
$V_{0.5}$ (mV)	3	–87.5	–93.9	–81.4	–109	–111	–107	–93.9	–98.3	–90.0

Pore opening

parameter	notes	VSD-I mean	VSD-I low CI 95%	VSD-I high CI 95%	VSD-III mean	VSD-III low CI 95%	VSD-III high CI 95%	VSD-IV mean	VSD-IV low CI 95%	VSD-IV high CI 95%
$V_{0.5}$ (mV)	7	5.21	2.27	7.31	7.06	3.43	12.9	–0.666	–2.48	1.41

Table S2. 4-state model kinetic parameters. Notes:
1: geometric mean
2: constrained parameter (charge conservation; eq.10)
3: calculated after fitting
4: fixed parameter (VSD-I only)
5: constrained parameter (microscopic reversibility; eq.9)
6: fixed parameter (all VSDs)
7: from Boltzmann fits of tail currents (eq.1) at V_h = –80 mV

TABLE S3

β_{2a}

	VSD-I (*n*=13 cells)			VSD-III (*n*=16)			VSD-IV (*n*=14)		
**Equilibrium constants at –80 mV**									
equilibrium	mean	low CI 95%	high CI 95%	mean	low CI 95%	high CI 95%	mean	low CI 95%	high CI 95%
R1↔A1	0.00681	0.00489	0.0113	0.00258	0.00161	0.00397	0.0261	0.0197	0.0340
R2↔A2	13.1	10.7	15.3	0.935	0.829	1.09	2.90	2.51	3.59
R1↔R2	0.0271	0.0209	0.0357	0.620	0.458	0.773	0.256	0.209	0.322
A1↔A2	52.1	33.5	85.8	225	133	331	28.4	20.8	39.1
**Equilibrium constants at 0 mV**									
R1↔A1	0.821	0.557	1.43	5.73	5.23	6.21	2.12	1.87	2.79
R2↔A2	1580	996	2600	79.4	53.9	123	30.1	23.8	37.9
R1↔R2	0.0271	0.0209	0.0357	16.2	8.38	27.3	2.00	1.27	3.28
A1↔A2	52.1	33.5	85.8	225	133	331	28.4	20.8	39.1

β_3

	VSD-I (*n*=10)			VSD-III (*n*=14)			VSD-IV (*n*=14)		
**Equilibrium constants at –80 mV**									
equilibrium	mean	low CI 95%	high CI 95%	mean	low CI 95%	high CI 95%	mean	low CI 95%	high CI 95%
R1↔A1	0.00495	0.00266	0.0111	0.00255	0.00159	0.00448	0.0207	0.0160	0.0282
R2↔A2	35.8	19.9	66.8	0.835	0.757	0.961	2.29	2.07	2.48
R1↔R2	0.0560	0.0385	0.0943	4.21	3.04	5.36	0.623	0.512	0.789
A1↔A2	405	132	752	1380	640	2500	68.6	48.2	99.7
**Equilibrium constants at 0 mV**									
R1↔A1	8.68	3.71	19.6	14.5	12.3	16.9	2.96	2.67	3.32
R2↔A2	62800	17100	261000	53.5	44.7	66.7	23.8	19.9	27.3
R1↔R2	0.0560	0.0385	0.0943	373	153	786	8.53	6.10	13.9
A1↔A2	405	132	752	1380	640	2500	68.6	48.2	99.7

Table S3. Transition equilibrium constants (quotient of forward and backward rates) at –80 and 0 mV.
