# Supplementary material for: A rich conformational palette underlies human CaV2.1-channel availability
Source: Nat Commun. 2025 Apr 23;16:3815. doi: 10.1038/s41467-025-58884-2 (PMC12019389; doi:10.1038/s41467-025-58884-2)
Supplement: Supplementary file 2 — Description of Additional Supplementary Files [file 41467_2025_58884_MOESM2_ESM.pdf]

Description of Additional Supplementary Files

File Name: Supplementary Movie 1

Description: An animated version of fig.6. The "state spectra" of the VSDs and the Pore (modeled after VSD-I) are at the top. At the bottom, the domains of the structure of the CaV2.1 pore-forming subunit (PDB: 8X90) are colored according to state occupancies and the holding potential.
